# Anthropogenic noise affects male house wren response to but not detection of territorial intruders

**DOI:** 10.1371/journal.pone.0220576

**Published:** 2019-07-31

**Authors:** Erin E. Grabarczyk, Sharon A. Gill

**Affiliations:** Department of Biological Sciences, Western Michigan University, Kalamazoo, MI, United States of America; University of Regina, CANADA

## Abstract

Anthropogenic noise decreases signal active space, or the area over which male bird song can be detected in the environment. For territorial males, noise may make it more difficult to detect and assess territorial challenges, which in turn may increase defense costs and influence whether males maintain territory ownership. We tested the hypothesis that noise affects the ability of male house wrens (*Troglodytes aedon*) near active nests to detect intruders and alters responses to them. We broadcast pre-recorded male song and pink noise on territories to simulate intrusions with and without noise, as well as to noise alone. We measured detection by how long males took to sing or approach the speaker after the start of a playback. To measure whether playbacks changed male behavior, we compared their vocal responses before and during treatments, as well as compared mean vocal responses and the number of flyovers and attacks on the speaker during treatments. Noise did not affect a male’s ability to detect an intruder on his territory. Males altered their responses to simulated intruders with and without noise compared to the noise-only treatment by singing longer songs at faster rates. Males increased peak frequency of songs during intrusions without noise compared to noise-only treatments, but frequency during intruder plus noise treatments did not differ from either. When confronting simulated intruders in noise, males increased the number of attacks on the speaker compared to intruders without noise, possibly because they were less able to assess intruders via songs and relied on close encounters for information. Although noise did not affect intruder detection, noise affected some aspects of singing and aggressive responses, which may be related to the challenge of discriminating and assessing territorial threats under elevated noise.

## Introduction

Selection on animal communication systems favors signalers that structure their signals to transmit with minimal degradation through the environment, as well as receivers with auditory systems capable of extracting relevant information from background noise [1, 2]. To elicit a response, a signal must first be detected, or separated from other sounds that make up background noise. Detection, however, is less likely to occur when background sounds occupying similar frequencies overlap with or mask the signal [3]. Sources of masking may be biological (calls of birds, anurans, or insects, e.g., [4]), geophysical (wind, rain, or thunder, e.g., [5, 6]), or anthropogenic (traffic, airplanes, or industrial generators, e.g., [7]) in origin. When background sounds are intermittent, animals may adjust the timing of their signals and vocalize during silent gaps [8–10]. But when sounds are continuous and masking is constant, the probability that receivers will detect a signal decreases. In particular, anthropogenic noise sources produce sounds that are often continuous, high amplitude, and low frequency [11]. Animals that vocalize at low frequencies experience more noise masking, which makes their signals harder to detect [12–14] and constrains communication [7].

The consequences of noise masking on vocal communication are wide ranging. Predation may increase if noise minimizes detection of alarm calls by adults [15, 16] or offspring [17]. If noise masks advertisement signals, males may fail to attract a mate, leading to reproductive failure [18]. Even if males succeed in attracting mates, noise masking could be costly for birds when they are defending active nests. Threats within or at the territory core pose greater risks to resident males compared to intruders on or near territory edges, as resident males risk losing mates, eggs, or nestlings if they are unable to repel an intruder [19]. Accordingly, males respond more aggressively to simulated intruders near the core part of their territory compared to territory edges by approaching more closely and increasing the number of flights towards the intruder [20]. Thus, if noise affects detection of and response to intruders around nests, males could suffer reproductive failure as well. However, little is known as to whether noise compromises intruder detection or response at active nests.

Whereas noise masking has the potential to lead to reduced fitness, the consequences of noise masking may be more subtle: noise could delay the detection of intruders and mask intruder signals thereby influencing the responses of males [21, 22]. In areas of chronic noise, territorial males respond more slowly to intruders: masking delays detection of an intruder’s song [21]. Once intruders are detected, how a male responds to them may influence whether he maintains ownership of his territory. In response to simulated intruders in quiet conditions, European robins *(Erithacus rubecula)* sing more low frequency notes, whereas in noisy conditions males eliminate low frequency notes [22] and decrease song complexity [23]. Song changes by male robins in noisy conditions may be perceived as less aggressive [23]. If noise modifies how intruders perceive territory holders, they may experience more intrusions and takeover attempts by other males.

We hypothesized that noise affects male responses to territorial intruders near their nests by decreasing the ability of males to detect intruders and by altering male responses to them. We tested this idea on male house wrens (*Troglodytes aedon*), a species in which neighbors sneak onto territories seeking extra-pair copulations [24] and non-territorial birds challenge resident males for nest sites and mates [25]. During such challenges, intruders often sing, which triggers resident males to chase intruders within their territory [26]. Takeover attempts have been reported in 10–13% of nesting attempts [25, 26] and occur when males are mate limited [26]. Risk of territorial takeovers vary across the season; early takeover attempts are more successful than those later in the season [25]. Nearly half of all challenges are successful, following which new territory holders typically kill the prior male’s offspring and re-initiate broods with the resident female [25]. During early breeding stages, males experience the added risk of paternity loss from intruders, whereas males during later stages risk loss of their entire reproductive investment. Accordingly, the responses of males to intruders may vary seasonally, and therefore we tested whether noise has similar effects on detection and responses to intruders near active nests early and late in the nesting cycle.

House wrens breed across a gradient of urbanization, and therefore likely experience variable noisy environments. Males adjust singing in response to fluctuations in noise, but social factors are important as well [27, 28]. In response to noise playback, paired males increase the peak frequency of their songs, whereas unpaired males do not [27]. In response to naturally varying noise, males decrease minimum frequencies of their songs, but only when breeding partners are fertile (Authors, unpublished data). Finally, despite variation in ambient noise and artificial light levels, nesting stage and the number of conspecific male neighbors predicts the onset of dawn singing in males [28]. Thus, noise may affect responses of males to territorial intruders, but these responses may be modified by social factors.

To test our hypothesis, we presented three treatments to breeding male house wrens: 1. pre-recorded song with pink noise to simulate intrusions with anthropogenic noise, 2. pre-recorded song without noise to simulate intrusions under natural ambient conditions, and 3. noise playback, to control for any confounding effects of pink noise playback. We tested color-banded males either prior to clutch initiation (early) or during incubation (late) at primary nests. During trials, we recorded male vocal responses and quantified movement behavior around the playback speaker. We determined whether noise affected intruder detection, whether playbacks changed male behavior, and finally, whether males responded differently to intruders depending on the presence of noise.

## Methods

### Study site and species

We studied a color-banded population of house wrens breeding in nest boxes at four urban to peri-urban natural areas in Kalamazoo County, Michigan, USA (42.290 N, 85.586 W). We arranged nest boxes (*n* = 108) in open habitat near forest edge, where house wrens prefer to nest. We checked nest boxes every three days to monitor use and breeding activity. We determined if a nest box was occupied by a male if sticks were present and he was singing to attract a mate. We considered a male paired once lining was added to the nest cup or we observed a female nest building. House wrens in our population typically lay 5–7 eggs (mean ± SD: 5.9 ± 1.0; EEG, unpublished data); therefore, we waited at least 1 day after clutches were completed to test males during the incubation stage. We captured adult house wrens by mist net and banded them with a USGS numbered aluminum band and 3 plastic bands for individual identification. We sexed individuals by cloacal protuberance (male) or presence of a brood patch (female), and confirmed sex by observing singing. Of the 45 males included in this study, 35 were banded, including 28 banded before experiments and seven banded 3–16 days after experiments but within the same breeding attempt. During mist netting, we first attempted to capture males without playback. If we were unsuccessful, we broadcast male or female house wren song recorded from a different population. Songs played during capture attempts were different from those used as experimental playback stimuli.

### Playback recordings

To create playbacks simulating a conspecific intruder, we selected songs recorded from 28 male house wrens during 2015–2016 in six natural areas in southwest Michigan. We recorded males during the dawn chorus using a Wildlife Acoustics Song Meter 2 (SM2) recording unit (Maynard, MA, 44.1 kHz, 16-bit sample rate, .wav format), connecting the SM2 microphone to the unit with a 10-m cord and then attaching the microphone to the tops of nest boxes. Although house wrens have large repertoires with an unknown number of song types [29], they sing with eventual variety [29, 30], repeating the same song type multiple times before gradually switching to a new type. Therefore, for song playback simulating a male intruder, we used a single song type, and repeated the same song every 15-sec for 10-min (approximately 4 songs/min), which compares to natural singing rates of males prior to clutch initiation in our populations (mean ± SD: 3.47 ± 2.0 songs/min*; n* = 20 males recorded from 630–730 (EST)). We selected songs for playback if they represented a unique song type, had a high signal-to-noise ratio, and fell within the range of song averages for duration and peak frequency (male song prior to clutch initiation *(n* = 1,124 songs from 45 males); duration, mean ± SD: 2.2 ± 0.5 s; peak frequency, mean: ± SD: 3.9 ± 0.6 kHz, EEG, unpublished data; playback duration, mean ± SD: 2.2 ± 0.4 s; peak frequency, mean ± SD: 4.0 ± 0.8 kHz). Song types or exemplars were defined as unique combinations of introduction and terminal note types (EEG, unpublished work). For each playback, songs were filtered in the waveform window (bandpass 1.3–11 kHz) to remove high- and low-frequency sounds that did not overlap male songs. We standardized song peak amplitudes with the amplify function (did not allow for clipping) in Audacity v2.1.2 in order for all songs to play at the same volume. During trials, we randomly selected a song playback until all 28 exemplars were played once before resampling from all possible playbacks *(n* = 45 trials). Individual focal males received the same song exemplar during treatments simulating an intruder with and without noise.

Noise playback experiments vary in the type of noise stimuli presented; some studies have broadcast pre-recorded traffic with peak amplitudes standardized across recordings (e.g., [31–33]), whereas others synthesize a constant noise signal (e.g., [12, 34–36]). Using pre-recorded traffic noise introduces complexity into field experiments as the focal bird may respond to amplitude peaks from passing vehicles as opposed to increased ambient SPLs [37]. To create a noise playback that mimics continuous anthropogenic noise, we created a pink noise signal, which like traffic has energy concentrated at 0–2 kHz that gradually decays with increasing frequency. We synthesized a 10-min pink noise signal in Avisoft SASLab Pro v 5.2 (R. Specht, Berlin, Germany; 44.1 kHz sample frequency, lowpass 1/f, frequency cut off at 0.20 Hz) and added 5-sec of fade in at the start of the signal to minimize startling focal males at the onset of the playback. Prior to field experiments, we measured the amplitude of males singing at their nest boxes with a SPL meter (76 dB fast averaging at 1 m, American Recorder Technology SPL-8810, EEG, unpublished data) and then determined the speaker volume necessary to play noise and male song and applied this setting to all males. We selected an amplitude of 76 dBA for noise playbacks because noise played at higher amplitudes (> 80 dBA) completely masks male songs, which prevents extraction and analysis of song traits, but noise played below 76 dBA may not change the ambient noise environment [35]. As a reference point, at 24 recording points on an urban campus with microphones positioned near roads and a major thoroughfare, the logarithmic mean SPL was 52.6 dBA (i.e. A-weighted dB) and logarithmic mean maximum SPL was 76.8 dBA (Gill et al., unpublished data). Therefore, pink noise played at 76 dBA is higher in amplitude than would be expected of typical continuous ambient SPLs in an urban landscape. Playback amplitude exceeded full spectrum SPLs measured on the focal male territories prior to playback (mean ± SD: 55.1 ± 7.8 dB, range = 37.1–62.7 dB, note that SPLs are measured in dB, rather than dBA, in Avisoft).

### Playback experiments

We performed focal male experiments (*n* = 45 males; 19 prior to clutch initiation (8 unpaired and 11 paired) and 26 during incubation) between sunrise and 1100 (EST) between June 6 –July 21, 2016 and April 28 –July 2, 2017. To simulate a male intruder, we played song from an amplified SME-AFS speaker (Saul Mineroff Electronics, New York) placed 5 – 10m from the focal male’s nest box, positioned 0.5–1.5m above ground in vegetation. To simulate a noise disturbance on the focal male’s territory, we broadcast noise from a second speaker placed approximately 10 m in the opposite direction of the nest box on the ground. We separated the noise playback speaker from the song playback speaker because we wanted to document whether song structure changed in response to the treatments. Males were attracted to the speaker playing song and if the speaker playing noise was nearby or noise was played from the same speaker as song, noise would mask the songs by focal males, making it impossible to extract song frequency [35]. Moreover, singing male birds move away from intense noise sources (SAG, unpublished data), such that the separation of intruder and noise may be more likely to mimic the location of an actual intruder relative to a noise source in the territory. To record focal male songs, we placed a Song Meter 2 recording unit (44.1 kHz, 16-bit sample rate, .wav format) between the nest box and speaker simulating an intruding male. To minimize disturbance during trials, we attached an Apple iPod (Cupertino, CA) using 20 m extension cords to each speaker and controlled onset of experiment at a distance.

We randomly selected the order of treatments to present. Each treatment consisted of a 10-min control period without playback followed by a 10-min treatment with a 10-min break between successive treatments. We chose to present all playback treatments on the same day, rather than on different days as male singing behavior changes as breeding progresses [29, 35, 38]. During trials, we quantified two behavioral responses: the number of times a male physically attacked the speaker (attack) and the number of times a male flew over the speaker (fly over), as well as the time (s) from start of playbacks for males to approach within 2 m of the speaker broadcasting intruder song. If males did not approach the speaker within 2 m during trials we entered approach time as 10-min plus one second in our analysis. From our recordings, we measured focal male vocal response time, or the time (s) from the beginning of a playback (song playback for intruder with and without noise, or the start of noise playback for noise alone) until a male sang.

### Acoustic analysis

We used a bandpass filter (1.3–11 kHz) to remove high and low frequency sounds from our recordings that did not overlap with male songs. In Avisoft, we used section labels to mark every focal male and intruder playback song recorded on the spectrogram window (Flat top window, 512 FFT length, 93.75% overlap, 0.725 ms time resolution). We quantified vocal responses of focal males to playbacks as rate of singing (song/min), song duration (s) and peak frequency (Hz), with the latter measured using automated parameter window, and compared vocal and behavioral responses for each 10-min control and stimulus. We analyzed song duration and peak frequency analysis only if songs were not overlapped by songs of another bird or playback. Across trials, we analyzed 8,495 focal male songs (during the control mean ± SD: 25.3 ± 22.9 songs per male and experimental playback periods mean ± SD: 36.1 ± 25.3 songs per male). Due to naturally occurring high ambient noise (which often exceeded song amplitudes) at some recording locations, we were unable to extract minimum frequency using automated parameter measures (see [35]). Avisoft consistently measured minimum frequency as the noise floor (i.e. the frequency of the high-pass filter) rather than the lowest frequencies of songs of focal males. We did not increase the cut-off frequency of the high-pass filter setting, as this adjustment would have eliminated the lowest frequency portions of focal male song.

To test whether ambient noise levels affected male response to treatments, we extracted sound pressure levels (SPLs) from focal male recordings. Each microphone and SM2 unit pair was calibrated with a Larson Davis CAL 200 sound level calibrator (Depew, NY) by recording a 1 kHz tone played at 94 dB. From the calibrated tone, we set the relative amplitude to 0 dB (re 20 μPa) in Avisoft for each recording. SPL measurements were taken from the first 10-min of focal male recordings by randomly selecting and averaging five 1-sec full spectrum noise samples that were not overlapped by house wren song.

### Statistical analysis

#### Detection

We used R program software v3.3.3 for all statistical analyses. To test whether treatments affected focal male response time, we compared how long males took to vocally respond after the start of each playback treatment and approach (s) the playback speaker within 2 m. We ran a linear mixed effects model using the package lme4 [39] to test if response time differed among treatments and included treatment, breeding stage, and the interaction between treatment and breeding stage as fixed effects and male identity and song exemplar as random effects. We approximated p-values with the package lmerTest [40] using a Satterthwaite approximation for hypothesis testing. Because we presented successive treatments with only 10-min breaks between them, an earlier treatment could have influenced a later one (i.e. a carry-over effect); therefore, for all models (detection and response), we initially included sequence of playback as an additional fixed effect. If playback sequence was significant predictor of male response, we could not isolate behavioral responses to the current treatment alone from the influence of an earlier treatment. We found sequence to be a significant predictor of male responses to our treatments (S1 and S2 Tables); therefore, we reanalyzed all models using only the first playback treatment and eliminated the second and third treatments for each male. Focal males did not approach the intruder playback speaker during any noise-alone trials, therefore we excluded this treatment from models exploring approach latency. Based on residual plots, the data showed heteroscedasticity, therefore we log transformed response time (vocal response time and approach latency) for the final model, which eliminated patterns of heteroscedasticity [41]. Male response time could have been influenced by ambient noise conditions; therefore, to explore whether ambient noise conditions influenced male detection, we plotted model residuals response time (s) against ambient noise (dB). We found no patterns that would indicate a relationship between noise and unexplained variation in the response time model.

#### Male responses to treatments

If males recognized the song playback as an intruder, we expected a change in vocal behavior from the pre-playback to the playback period. To demonstrate that playbacks altered male singing, we calculated the mean difference between song traits in the 10-min control period without playback from 10-min playback stimulus average. To determine if treatment affected male response during playback, we calculated mean responses for song rate (song/min), song duration (s), and peak frequency (Hz) during each 10-min playback treatment. We used linear mixed effects models to test whether males changed their songs differently depending on treatments (i.e. before versus during treatments) and whether mean song traits produced during trials differed among treatments. We included treatment, breeding stage, and the interaction between treatment and breeding stage as fixed effects, and male identity and song playback exemplar as random effects. We compared model fit with and without interaction terms using AICc values and ΔAICc. Models with a change in AICc of 2 or less were selected for analysis. If models with and without the interaction term did not differ, we selected the model without the interaction term for analysis (Table 1). We detected a carry-over effect in male song length and rate of singing to subsequent treatments, therefore to be consistent, we analyzed only song traits (song length, rate, and peak frequency) from the first treatment presented to each male. We used residual plots to test model adequacy and ran pairwise comparisons with the package lsmeans [42] to determine whether song traits differed between treatments. For each model, we plotted residuals against year tested (2016 or 2017), Julian date, and ambient noise, but found no dependencies for these factors and did not include any in final models. During initial data exploration, we used box plots to assess variation in responses of paired versus unpaired males prior to clutch initiation; these plots revealed little or no differences in behavior between paired and unpaired males in our sample and therefore we did not include pairing status in our models. We also explored whether treatment affected the proportion of focal male songs overlapped by the playback recording or playback songs that overlapped the songs of focal males. Data exploration plots showed no patterns that suggested the proportion of overlapped and non-overlapped songs differ due to treatment.

**Table 1:** Comparison of competing models for change in male response and mean response with and without the interaction term using AICc and ΔAICc.

Analysis	Model	AICc	ΔAICc
Change in peak frequency (Hz)	Main effects	509.51	0.00
	Interaction	511.99	2.48
Change in song duration (s)	Main effects	28.68	0.03
	Interaction	28.71	0.00
Change in song rate (songs/min)	Main effects	225.06	0.00
	Interaction	228.67	3.61
Mean peak frequency (Hz)	Main effects	606.55	0.00
	Interaction	609.91	3.36
Mean song duration (s)	Main effects	30.52	0.18
	Interaction	30.34	0.00
Mean song rate (songs/min)	Main effects	223.78	0.00
	Interaction	226.19	2.41

In addition to vocal traits, change in male response to an intruder could also include movement behavior such as fly overs or attacks near the playback speaker. For non-vocal behavior during trials, we used a permutation test of independence to compare the number of fly overs and attacks during a simulated intruder with and without noise. In addition, we used a Fisher exact test to determine whether the proportion of males that responded during trials with either fly overs or attacks differed between simulated intrusions with and without noise. During playbacks with noise only, we recorded no fly overs or attacks from any focal male tested, thus eliminated this treatment from analysis. Based on data plots, sequence of presentation was significant, therefore we compared the number of fly overs and attacks to the first playback treatment during an intrusion (*n* = 14) and to an intruder with noise (*n* = 12).

### Ethics statement

All experiments were approved by Western Michigan University’s Institutional Animal Care and Use Committee (IACUC No. 16-01-01). Permission to use study sites for experiments was granted by the Asylum Lake Council, Western Michigan University, and the Southwest Michigan Land Conservancy. Permission to band birds was granted by the USGS (Federal Bird Banding Permit No. 23665) and by the Michigan Department of Natural Research Scientific Collectors Permit (SC 1394).

## Results

### Noise did not delay intruder detection

**Fig 1:**
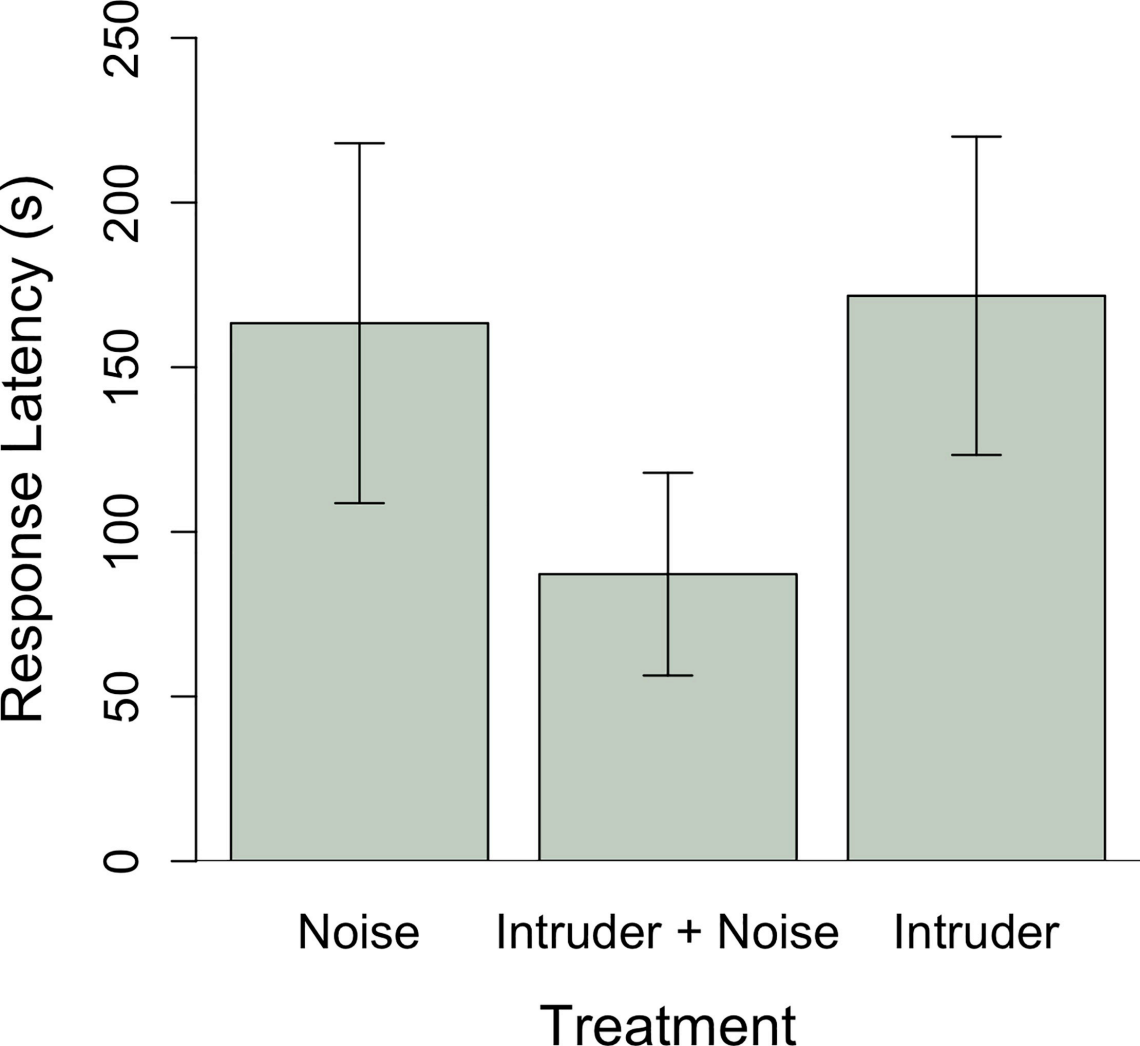
Male house wrens did not differ in the delay from beginning of playbacks to their first songs based on treatment (*n* = 12 intruder alone, *n* = 15 intruder plus noise, *n* = 18 noise alone).

If noise masks intruder signals resident males may be slower to detect intruders on their territories. We found treatment had no effect on intruder detection measured as the duration from start of first playback to a male’s first song (intruder treatment: Estimate ± SE: 0.8 ± 0.6, t = 1.3, p = 0.2; intruder + noise treatment: Estimate ± SE: 0.07 ± 0.5, t = 0.1, p = 0.9; Fig 1). Males during early and late breeding stages sang in response to intruders with similar latency (stage early: Estimate ± SE: –0.9 ± 0.5, t = -1.6, p = 0.1). Similarly, males may be slower to approach an intruder if noise disrupted detection. However, treatment did not affect how quickly males approached the playback speaker within 2 m (intruder + noise treatment: Estimate ± SE: -0.3 ± 0.5, t = -0.5, p = 0.6). Breeding stage was not a significant predictor of a male’s latency to approach the playback speaker (stage early: Estimate ± SE: -0.9 ± 0.6, t = -1.6, p = 0.1).

### Males change vocal behavior before and during treatments

**Fig 2:**
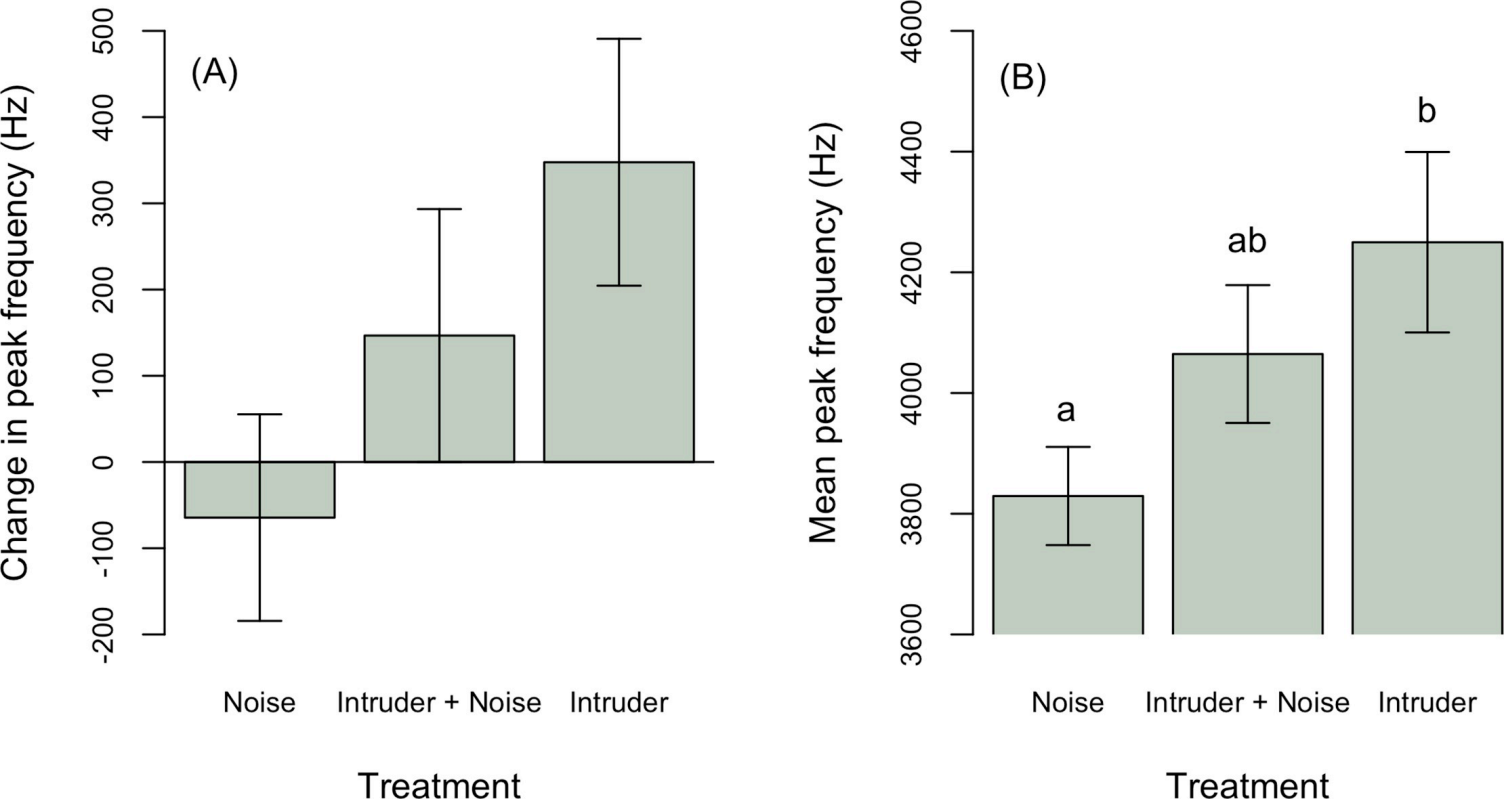
In response to an intruder, (A) male house wrens increase song peak frequency (Hz) during an intrusion with (*n* = 13) and without noise (*n* = 12), and but decreased peak frequency in response to noise alone (*n* = 8) compared to pre-playback control periods. (B) On average males sing at a higher peak frequency during an intrusion without noise (*n* = 13) compared to the noise only treatment (*n* = 14). Male responses to an intruder with noise (*n* = 13) did not differ from either the noise only or intruder only treatment. Breeding stage was not a significant predictor of song peak frequency.

**Fig 3:**
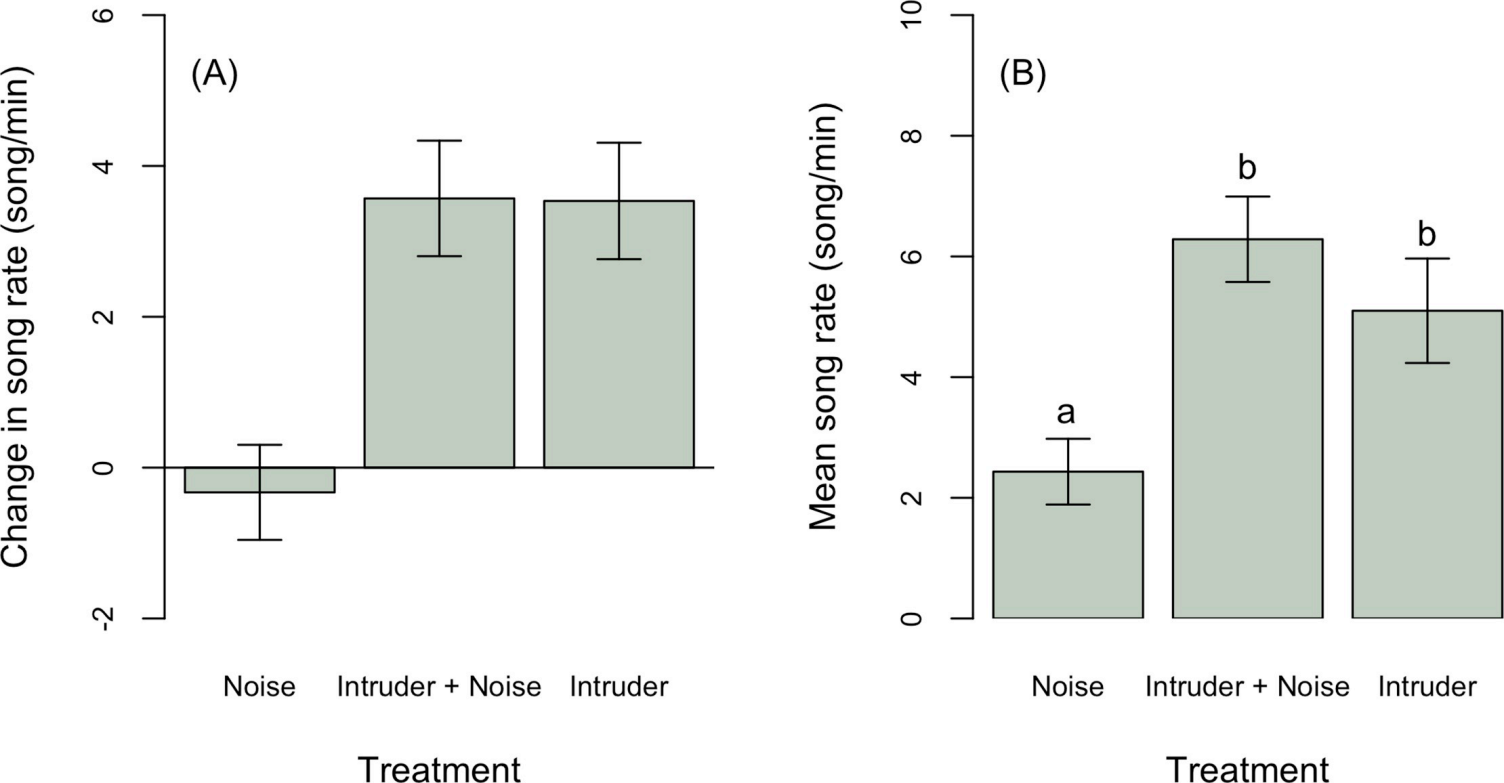
Male house wrens increased singing rate (A) in response to an intruder with (*n* = 12) and without noise (*n* = 15), but they did not change singing rate in noise (*n* = 18) compared to pre-playback control periods. (B) On average males sang at a higher rate when an intruder was present, regardless of whether or not noise was played, than during noise playback alone.

**Fig 4:**
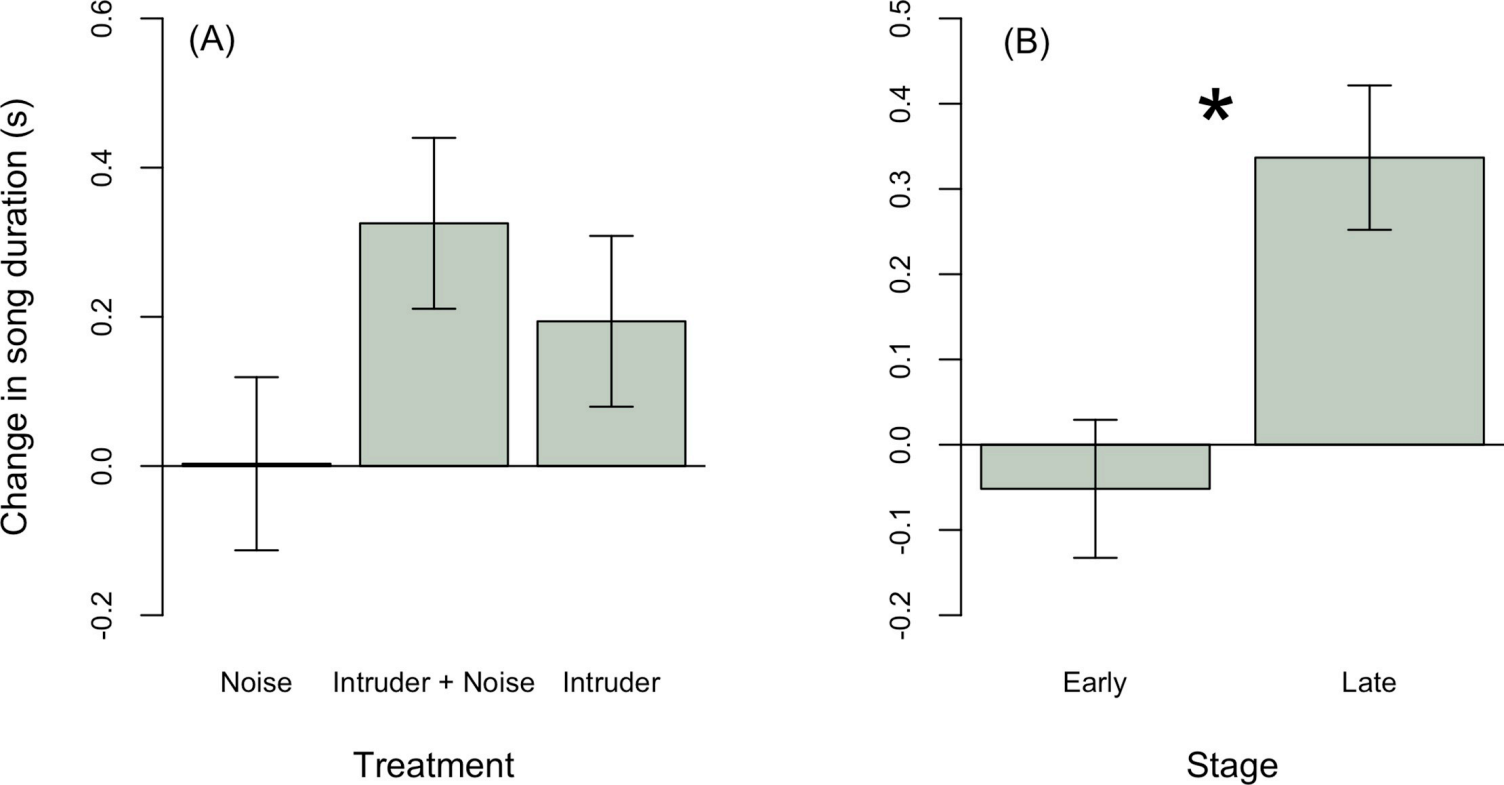
(A) Treatment did not influence change in song duration. (B) During later breeding stages, males increased their song duration in response to treatments (*n* = 13 intruder alone, *n* = 12 intruder with noise, *n = 14 noise alone*), whereas males during early stages overall did not increase their song duration.

To explore the effect of playbacks on male behavior, we first tested whether males changed their songs from the pre-playback to the playback period. Overall, males increased song peak frequency in response to intruders (Estimate ± SE: 439.8 ± 223.0, t = 1.9, p = 0.06, Fig 2A), although change in song peak frequency did not statistically differ among treatments (Table 2). Males increased song rate, producing on average 4 songs more per minute in response to a simulated intruder, regardless of noise (intruder only: Estimate ± SE: 3.4 ± 1.0, t = 3.4, p = 0.002; intruder + noise treatment: Estimate ± SE: 3.7 ± 2.0, t = 3.8, p = 0.0005), and on average slightly decreased song rate in response to noise alone (Fig 3A). Males in early breeding stages sang longer songs (stage early: -0.4 ± 0.1, t = -3.5, p = 0.001; Table 2, Fig 4B), but treatment had no effects on song duration (Fig 4A).

**Table 2:** Change in song trait by male house wrens in response to a simulated intruder with and without noise, and to noise alone.

Analysis	Parameter	Estimate ± SE	t_(df)_	p-value
Change in peak frequency (Hz)	Intercept	–99.8 ± 194.8	–0.5_(24)_	0.6
	Treatment: intruder	439.8 ± 223.0	1.9_(29)_	0.06
	Treatment: intruder + noise	215.2 ± 221.8	1.0_(29)_	0.3
	Breeding stage: prelaying	63.6 ± 180.2	0.4_(29)_	0.7
Change in song duration (s)	Intercept	0.2 ± 0.1	1.7_(29)_	0.1
	Treatment: intruder	0.1 ± 0.1	0.6_(25)_	0.5
	Treatment: intruder + noise	0.3 ± 0.2	1.9_(28)_	0.06
	Breeding stage: prelaying	–0.4 ± 0.1	–3.5_(27)_	0.001
Change in song rate (songs/min)	Intercept	0.6 ± 0.8	0.7_(41)_	0.5
	Treatment: intruder	3.4 ± 1.0	3.4_(37)_	0.002
	Treatment: intruder + noise	3.7 ± 2.0	3.8_(38)_	0.0005
	Breeding stage: prelaying	–1.7 ± 0.8	–2.0_(37)_	0.05

### Males respond differently to simulated intruders depending on noise

**Fig 5:**
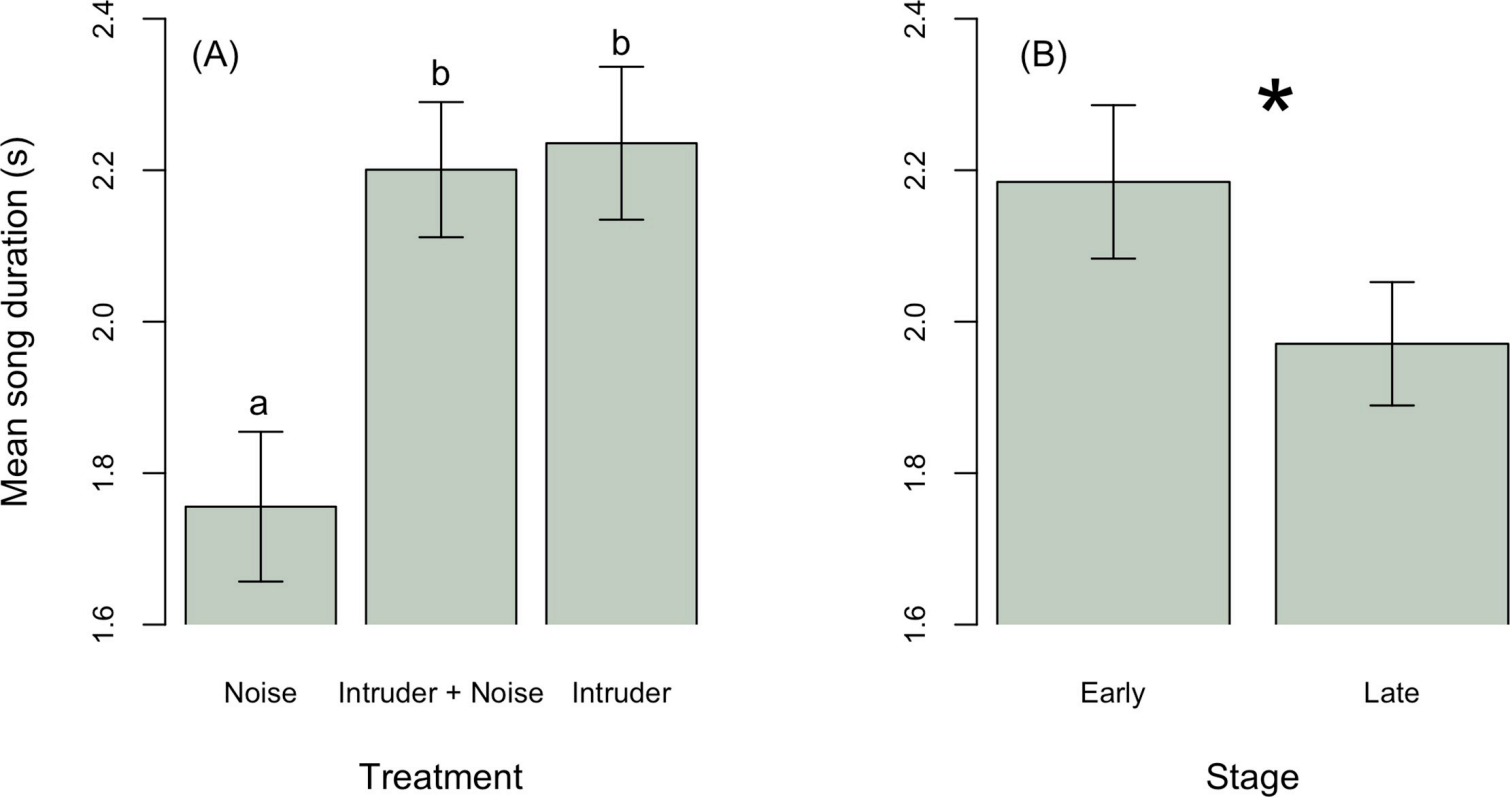
(A) Mean song duration was longer in response to an intruder with (*n* = 13) and without noise (*n* = 13) compared to noise alone (*n* = 14). (B) During early breeding stages (*n* = 16) males sang longer songs compared to later stages (*n* = 24).

If noise affects how territorial males respond to intruders, then the average vocal response of males should differ between intrusions with and without noise. Males sang on average at a higher peak frequency during intruder playback without noise compared to treatments with noise alone (Fig 2B, Table 3). Average peak frequency responses to an intruder with noise did not differ from either the noise only or intruder only treatment, suggesting male responses are intermediate between intruder only and noise only treatments (Fig 2B). Breeding stage did not affect mean song peak frequency during treatments (Table 3). Males sang longer songs (Fig 5A) at a higher rate (Fig 3B) in response to intruders regardless of noise compared to noise alone (Table 3). Males sang longer songs regardless of treatment during early compared to late breeding stages (Fig 5B). Breeding stage did not affect mean song rate.

**Table 3:** Mean song trait response by male house wrens in response to a simulated intruder with and without noise, and to noise alone.

Analysis	Parameter	Estimate ± SE	t_(df)_	p-value
Mean peak frequency (Hz)	Intercept	3831.5 ± 136.4	28.1_(36)_	<0.00001
	Treatment: intruder	432.3 ± 168.1	2.6_(34)_	0.01
	Treatment: intruder + noise	253.1 ± 166.9	1.5_(36)_	0.1
	Breeding stage: prelaying	–48.0 ± 142.3	–0.3_(36)_	0.7
Mean song duration (s)	Intercept	1.6 ± 0.1	15.8_(36)_	<0.00001
	Treatment: intruder	0.5 ± 0.1	4.4_(33)_	0.0001
	Treatment: intruder + noise	0.4 ± 0.1	3.6_(36)_	0.001
	Breeding stage: prelaying	0.3 ± 0.1	2.6_(35)_	0.015
Mean song rate (songs/min)	Intercept	1.8 ± 0.8	2.3_(41)_	0.03
	Treatment: intruder	3.1 ± 1.0	3.0_(41)_	0.003
	Treatment: intruder + noise	4.0 ± 1.0	4.0_(41)_	0.0002
	Breeding stage: prelaying	1.2 ± 0.8	1.4_(41)_	0.2

**Fig 6:**
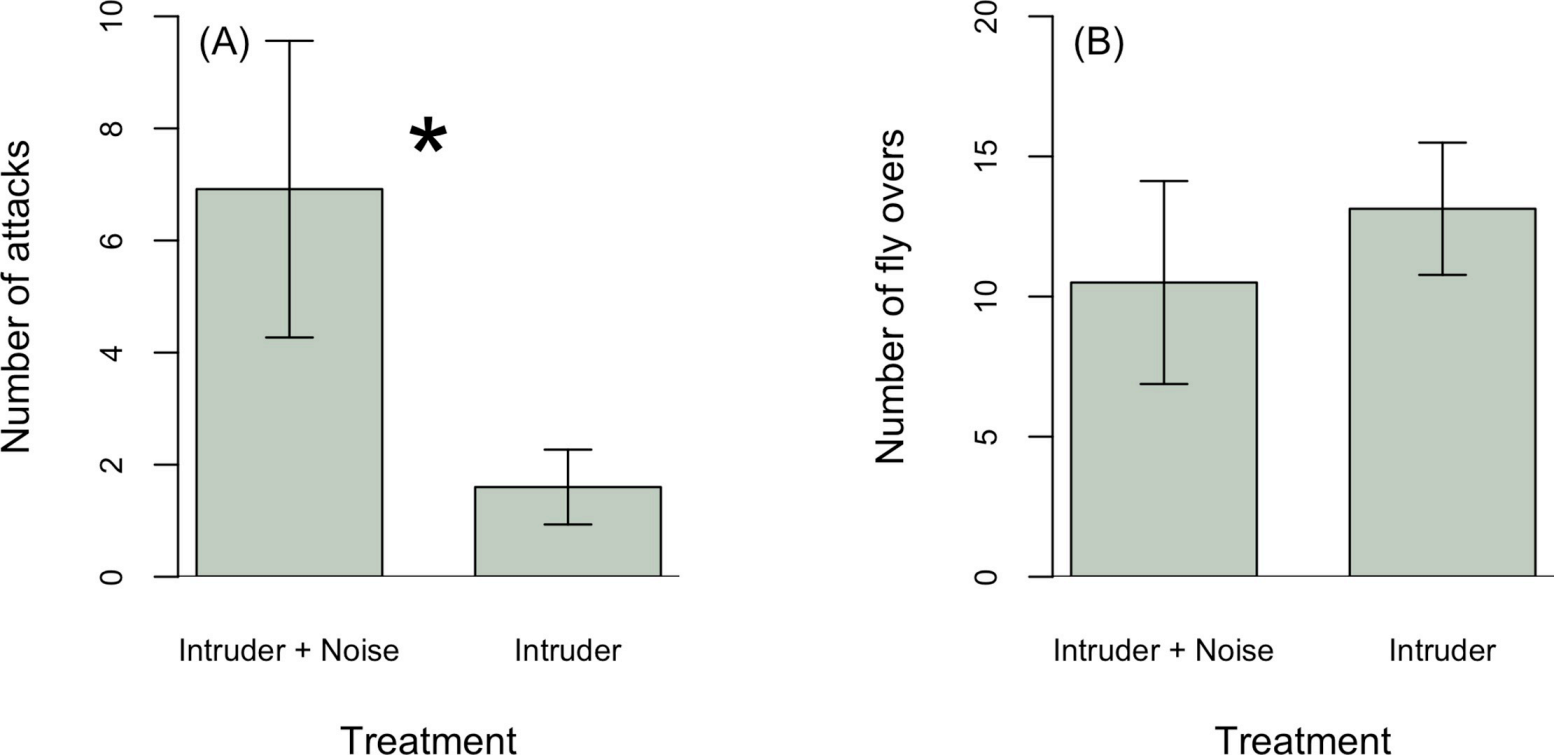
Males attacked the playback speaker more during an intrusion with noise (*n* = 14) compared to an intruder alone (*n* = 12; (A)), treatment did not affect the number of fly overs towards the speaker (B).

To test whether males differed in their non-vocal response towards the speaker during a simulated intrusion with and without noise, we compared the total number of fly overs and attacks on the speaker during each treatment. For analysis, we included fly over and attack data for only the first treatment presented to each male in response to an intruder (*n* = 14) without and with noise (*n* = 12). Males attacked the playback speaker significantly more times in response to an intruder with noise compared to without noise (Z = –2.01, p = 0.04, Fig 6A). The number of male fly overs did not differ between treatments (Z = 1.1, p = 0.3, Fig 6B). The proportion of males to attack or fly over the speaker during simulated intrusions with and without noise did not differ (Fisher exact test: attack, p = 0.5; fly over, p = 0.6). Of the 27 males included in analysis, 40.7% (*n* = 11) attacked the playback speaker during song treatments (*n* = 6 out of 12 males during intrusion with noise and *n* = 5 out of 14 males during an intrusion without noise). Of the 26 males, 84.6% of males (*n* = 22) responded by flying over the speaker.

## Discussion

Anthropogenic noise masking reduces the active space of male long-distance signals used for mate attraction and territory defense [27, 43]. Noise may be particularly costly to males when defending territories if they are less able to detect territorial intruders or if noise alters their ability to assess intruders. We tested the hypothesis that anthropogenic noise affects male house wrens’ detection of and responses to conspecific territorial intruders near active nests. Males first sang and approached the speaker with similar delay from the onset of playbacks regardless of noise, suggesting that noise treatments did not affect the ability of males to detect territorial intruders. However, during simulated intrusions paired with noise playback, males attacked the playback speaker more compared to intrusions without noise, suggesting that noise alters aggressive responses to intruders. Males sang longer songs more often to simulated intruders regardless of noise playback, compared to the noise alone. Males increased song peak frequency in response to intruders. Responses to an intruder with noise were intermediate between the noise only and intruder only treatments, suggesting that noise did not compromise vocal responses to intruders, but did dampen male peak frequency responses.

Under high ambient noise conditions, resident males could be delayed or fail to detect intruders on their territory if noise alters the probability of intruder detection. With increasing ambient noise, spotted towhees (*Pipilo maculatus*) and chipping sparrows (*Spizella passerina*) more slowly approach a playback speaker broadcasting intruder song [21], whereas Nuttall’s white-crowned sparrow (*Zonotrichia leucophrys nuttalli*) approach more quickly [44]. The differences in response time may be attributed to where the playback took place on the male’s territory, as Phillips and Derryberry [44] explicitly identified focal male territories and simulated intrusions at the territory core, whereas Kleist et al. [21] did not identify territory boundaries. By contrast, noise did not influence response time by male house wrens, as the time elapsed from start of playback to first song and latency to approach the playback speaker did not differ by treatment. We performed playback experiments near active primary nests, which may have facilitated detection by males regardless of treatment, as males are more likely to spend time at or near active nests during the breeding season.

Noise could affect the ability of territorial males to adequately assess the threat posed by intruder. Such an effect could occur if noise masks intruder signals, resulting in more intense responses under noisy conditions. During simulated intrusions with noise, focal males attacked the speaker more frequently compared to intrusions without noise (Fig 6A). Our results are similar to patterns found in other birds, which respond aggressively by approaching intruders more closely with increasing ambient noise levels, likely enabling males to better discriminate the threat [44, 45]. During actual intrusions, closer approaches and increased attacks on or towards an intruder might lead to interactions escalating more quickly, which could be physically costly to both participating males. However, less than half of the males in our study responded by physically attacking the speaker during playbacks, and only five out of 14 males responded with attacks during a simulated intrusion without noise. This result, based on simulated intrusions with playbacks, differs from interactions with actual intruders in which males immediately approach and attack intruding males [25]. In response to actual intrusions, males may also rely on visual cues and movements in addition to acoustic detection as part of their territorial response.

During intrusions, the information conveyed via signals to neighboring birds may be important if neighbors eavesdrop thereby passively gaining information about aggressive encounters. Male house wrens increased song peak frequency during intrusions regardless of noise, but male responses to an intruder with noise were intermediate between the noise only and intruder only treatments (Fig 2B). This pattern suggests noise may weaken male frequency responses to a territorial intruder. Song frequency adjustments to intruders could be an indication of aggressive intent [46] and may reflect signaler body size or condition [47]. The degree to which males adjust song frequency during intrusions may affect their reproductive success if females eavesdrop on territorial interactions and use information regarding male performance for mate choice decisions [48, 49]. Neighboring males might also eavesdrop on territorial interactions and use information gained passively to guide their own responses to territorial intruders [50].

Longer songs given at high rates improve the likelihood of detection by increasing redundancy [51], but could also be used as an aggressive signal to overlap with an intruders’ song [52]. Focal males sang longer songs at higher rates during early breeding stages compared to later ones, a finding consistent with prior studies [35, 38, 53]. However, these early breeding males did not adjust song duration to playbacks, whereas males at later breeding stages sang longer duration songs in response to treatments, possibly reflecting greater investment by males in defense of offspring that are closer to fledging (e.g., [54]). During early stages of breeding, male songbirds broadcast mate attraction signals at high rates that transmit over large distances and are locatable by females, but also function to repel conspecific male competitors [19]. For house wrens, song duration may therefore play an important role in both territorial defense and mate attraction signaling. In addition to repelling intruders, focal males may adjust temporal song traits to mask or overlap their challenger’s signal. By simply increasing song rate and duration, the probability of overlap also increases. However, further testing is necessary to determine whether song rate and duration adjustments by intruders elicit a gradient of responses by focal males. This, in combination with evidence males adjust the timing of their signals relative to playback, could be evidence of an aggressive signal in response to territorial intruders.

Anthropogenic noise pollution is a widespread and increasingly common feature in urban natural areas. For birds, we might expect that species inhabiting noisy areas are those that are able to adjust their behaviors such that they reduce the cost of breeding in noise [55, 56]. In this study, we used playback simulations to test whether experimental noise altered territorial male responses to an intruder around active nests. Importantly, we provide additional support that at territory cores, noise does not delay intruder detection [44]. Masking may hinder the ability of males to discriminate intruder signals, as suggested by the closer approaches (see [44]) and an increased number of attacks (this study) elicited by playbacks in noise. Males show an intermediate song peak frequency response to an intruder with noise compared to the noise only and intruder only treatments, suggesting noise affects some aspects of singing. We show focal males adjusted their song length and rate to an intruder similarly in both quiet and noisy conditions. Spectral and temporal vocal adjustments in response to an intruder may increase the active space of focal male signals, which could be important if neighboring conspecifics eavesdrop to gain information on aggressive interactions. In summary, responses of male house wrens to intruders differed depending on noise, but were not completely compromised by noise. Presumably, noise has been a persistent disturbance within our study areas, and the responses we measured from males breeding in these established urban natural areas may be learned behaviors as a result of past environmental change, rather than maladaptive responses to a novel environmental disturbance [57].

## Supporting information

S1 Table Change in song trait by male house wrens in response to a simulated intruder with and without noise, and to noise alone. (DOCX)

S2 Table Mean song trait response by male house wrens in response to a simulated intruder with and without noise, and to noise alone. (DOCX)
