# Supplementary material for: Anthropogenic noise affects male house wren response to but not detection of territorial intruders
Source: PLoS One. 2019 Jul 31;14(7):e0220576. doi: 10.1371/journal.pone.0220576 (PMC6668836; doi:10.1371/journal.pone.0220576)
Supplement: S1 Table — (DOCX) [file pone.0220576.s001.docx]

**Supporting information**

**S1 Table** Change in song trait by male house wrens in response to a simulated intruder with and without noise, and to noise alone

| Analysis | Parameter | Estimate ± SE | t_(df)_ | p-value |
| --- | --- | --- | --- | --- |
| Change in peak frequency (Hz) – all treatments^a^ | Intercept | –91.8 ± 136.5 | –0.7_(104)_ | 0.5 |
|  | Treatment: intruder | 387.5 ± 107.0 | 3.6_(75)_ | 0.0005 |
|  | Treatment: intruder + noise | 290.9 ± 106.8 | 2.7_(75)_ | 0.008 |
|  | Breeding stage: prelaying | 216.2 ± 146.6 | 1.5_(97)_ | 0.1 |
|  | Sequence | -41.5 ± 42.9 | –1.0_(77)_ | 0.4 |
|  | Intruder*prelaying | –170.6 ± 173.5 | –1.0_(76)_ | 0.3 |
|  | Intruder + noise*prelaying | –318.5 ± 174.1 | –1.8_(77)_ | 0.07 |
| Change in song duration (s) – all treatments^a^ | Intercept | 0.2 ± 0.09 | 2.5_(93)_ | 0.01 |
|  | Treatment: intruder | 0.1 ± 0.06 | 2.2_(79)_ | 0.03 |
|  | Treatment: intruder + noise | 0.1 ± 0.06 | 1.7_(80)_ | 0.09 |
|  | Breeding stage: prelaying | –0.1 ± 0.06 | –2.1_(42)_ | 0.04 |
|  | Sequence | –0.1 ± 0.03 | –3.2_(80)_ | 0.002 |
| Change in song duration (s) – first treatment only^b^ | Intercept | 0.2 ± 0.1 | 1.7_(29)_ | 0.1 |
|  | Treatment: intruder | 0.1 ± 0.1 | 0.6_(25)_ | 0.5 |
|  | Treatment: intruder + noise | 0.3 ± 0.2 | 1.9_(28)_ | 0.06 |
|  | Breeding stage: prelaying | –0.4 ± 0.1 | –3.5_(27)_ | 0.001 |
| Change in song rate (songs/min) – all treatments^a^ | Intercept | 0.6 ± 0.7 | 0.9_(129)_ | 0.4 |
|  | Treatment: intruder | 3.9 ± 0.5 | 7.3_(107)_ | 0.0000 |
|  | Treatment: intruder + noise | 3.2 ± 0.5 | 5.9_107)_ | 0.0000 |
|  | Breeding stage: prelaying | 1.2 ± 0.5 | –0.4_(85)_ | 0.7 |
|  | Sequence | –0.6 ± 0.3 | –2.2_(107)_ | 0.03 |
| Change in song rate (songs/min) – first treatment only^b^ | Intercept | 0.6 ± 0.8 | 0.7_(41)_ | 0.5 |
|  | Treatment: intruder | 3.4 ± 1.0 | 3.4_(37)_ | 0.002 |
|  | Treatment: intruder + noise | 3.7 ± 2.0 | 3.8_(38)_ | 0.0005 |
|  | Breeding stage: prelaying | –1.7 ± 0.8 | –2.0_(37)_ | 0.05 |

^a^ We first analyzed all treatments presented to males and included treatment, breeding stage, and sequence of presentation as fixed effects, male identity and song exemplar as random effects.

^b^ For models where sequence was a significant predictor of the change in male response we eliminated the second and third treatments, and reanalyzed the model including only the first treatment presented.
