# Supplementary material for: Anthropogenic noise affects male house wren response to but not detection of territorial intruders
Source: PLoS One. 2019 Jul 31;14(7):e0220576. doi: 10.1371/journal.pone.0220576 (PMC6668836; doi:10.1371/journal.pone.0220576)
Supplement: S2 Table — (DOCX) [file pone.0220576.s002.docx]

**S2 Table** Mean song trait response by male house wrens in response to a simulated intruder with and without noise, and to noise alone

| Analysis | Parameter | Estimate ± SE | t_(df)_ | p-value |
| --- | --- | --- | --- | --- |
| Mean peak frequency (Hz) – all treatments^a^ | Intercept | 3878.3 ± 112.7 | 34.4_(105)_ | <0.00001 |
|  | Treatment: intruder | 221.7 ± 74.7 | 3.0_(85)_ | 0.004 |
|  | Treatment: intruder + noise | 201.7 ± 74.4 | 2.7_(85)_ | 0.009 |
|  | Breeding stage: prelaying | – 26.7 ± 85.9 | –0.3_(82)_ | 0.8 |
|  | Sequence | 33.8 ± 37.3 | 0.9_(55)_ | 0.4 |
| Mean song duration (s) – all treatments^a^ | Intercept | 1.9 ± 0.09 | 20.1_(73)_ | 0.000 |
|  | Treatment: intruder | 0.1 ± 0.05 | 2.4_(81)_ | 0.02 |
|  | Treatment: intruder + noise | 0.1 ± 0.05 | 2.7_(80)_ | 0.009 |
|  | Breeding stage: prelaying | 0.1 ± 0.07 | 1.6_(96)_ | 0.1 |
|  | Sequence | 0.03 ± 0.03 | 1.2_(80)_ | 0.2 |
| Mean song rate (songs/min) – all treatments^a^ | Intercept | 2.0 ± 0.7 | 2.9_(126)_ | 0.005 |
|  | Treatment: intruder | 2.3 ± 0.5 | 4.8_(87)_ | 0.0000 |
|  | Treatment: intruder + noise | 2.0 ± 0.5 | 4.0_(87)_ | 0.0000 |
|  | Breeding stage: prelaying | 1.0 ± 0.7 | 1.5_(43)_ | 0.1 |
|  | Sequence | 0.9 ± 0.2 | 3.6_(87)_ | 0.0004 |
| Mean song rate (songs/min) – first treatment only^b^ | Intercept | 1.8 ± 0.8 | 2.3_(41)_ | 0.03 |
|  | Treatment: intruder | 3.1 ± 1.0 | 3.0_(41)_ | 0.003 |
|  | Treatment: intruder + noise | 4.0 ± 1.0 | 4.0_(41)_ | 0.0002 |
|  | Breeding stage: prelaying | 1.2 ± 0.8 | 1.4_(41)_ | 0.2 |

^a^ Models describing mean song duration and peak frequency males included treatment, breeding stage, and sequence of presentation as fixed effects, male identity and song playback exemplar as random effects.

^b^ For models describing mean song rate during treatments sequence was a significant predictor therefore we eliminated the second and third treatments, and reanalyzed and interpret the model including only the first treatment presented.
